# Visual biofeedback for paradoxical vocal fold motion (PVFM)

**DOI:** 10.1186/s40463-021-00495-0

**Published:** 2021-02-18

**Authors:** Rachelle Alyce LeBlanc, Daniel Aalto, Caroline C. Jeffery

**Affiliations:** 1 Division of Otolaryngology Head and Neck Surgery, Department of Surgery, University of Alberta, 1E4 Walter Mackenzie Centre, 8440-112 Street NW, Edmonton, Alberta T6G 2B7 Canada; 2 Faculty of Rehabilitation Medicine, University of Alberta, Edmonton, Alberta Canada

**Keywords:** Paradoxical vocal fold motion, Biofeedback

## Abstract

**Objectives:**

Paradoxical vocal fold motion (PVFM) is a common condition where the vocal folds inappropriately adduct during inspiration. This results in dyspnea and occasionally significant distress. The condition is thought to be primarily functional, with behavioural therapy considered mainstay in the non-acute setting. However, practice variations and limited access to speech language pathology (SLP) services can pose management challenges. We aimed to examine the efficacy of surgeon performed visual biofeedback as first-line treatment for PVFM.

**Study design:**

Prospective, non-randomized, non-comparative clinical study.

**Methods:**

Adult patients referred for possible PVFM and congruent laryngoscopy findings over a two-year period were included. Patients were excluded if they presented in acute distress, had alternate diagnosis to explain symptomology and/or coexisting untreated lower respiratory pathology. Patients underwent immediate surgeon-performed visual biofeedback on the same visit day. The primary outcome of interest was change in Dyspnea Index (DI) scores pre- and post-intervention 3 months follow-up. The secondary outcome measured was change in asthma medication use from baseline to follow-up.

**Results:**

Of 34 patients presenting, 25 met inclusion criteria. Of these, 72% were female with an average age of 36.9 ± 14.1. Approximately 48% of patients had a diagnosis of well-controlled asthma at presentation and co-morbid psychiatric diagnoses were common (52%). Pre- and post-intervention analysis showed significant improvement in DI scores (*p* < 0.001) and reduction in bronchodilator use (*p* = 0.003).

**Conclusion:**

This is a prospective study that evaluates the role of visual biofeedback in PVFM patients. Our data suggests that visual biofeedback effectively reduces short-term subjective symptoms and asthma medication use.

**Level of evidence:**

3

**Graphical abstract:**

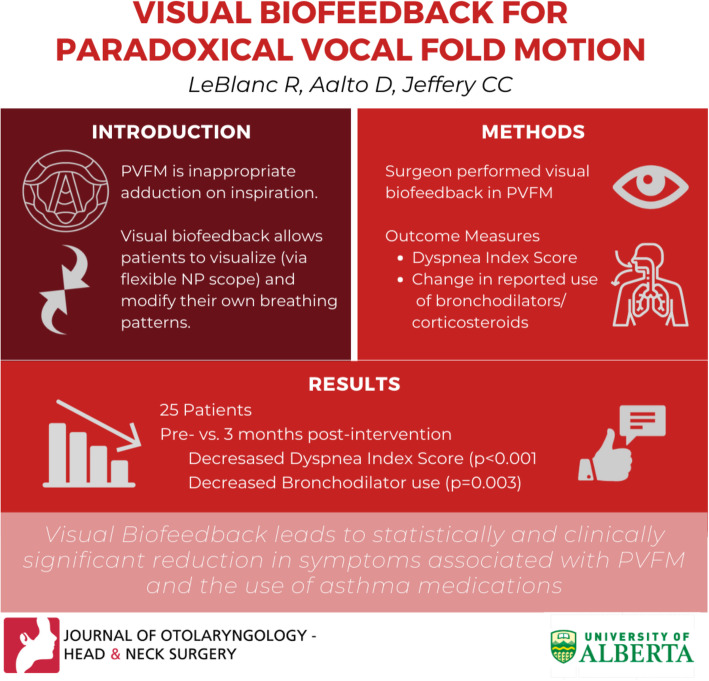

## Introduction

Paradoxical vocal fold motion (PVFM) or inducible laryngeal obstruction (ILO) is a common condition characterized by abnormal adduction of the vocal folds during inspiration [1, 2]. It is within the spectrum of conditions that includes laryngeal hypersensitivity and laryngospasm, where complete adduction and obstruction occurs [3, 4]. The term vocal cord dysfunction is also commonly used by non-otolaryngologists, but is a problematic term as the vocal folds have normal structure and innervation. The consequent airway obstruction symptoms include stridor, dysphonia, and cough [5]. PVFM is particularly common in women and is often mistaken for asthma, allergic reactions, vocal fold motion abnormalities, and croup [6]. Patients are often misdiagnosed and this often leads to unnecessary intubations, long-term medical and/or surgical therapy [6]. Proper diagnosis is key for avoiding unnecessary treatments.

PVFM is increasingly recognized as a functional disorder rather than psychogenic in etiology [7]. Impaired laryngeal sensitivity, inflammation, gastroesophageal reflux disease, exercise intolerance, and viral illness have all been proposed as potentiating factors described in the literature [7]. Patients usually present with a history of episodic upper airway symptoms, often with audible inspiratory or biphasic stridor, respiratory distress, inability to speak easily and severe anxiety [7, 8]. While laryngoscopy during a symptomatic episode is the considered the gold standard for diagnosis [1, 2], physical exam findings between episodes are generally normal. In the non-acute setting, laryngoscopy remains important to excluding any upper airway pathology and can be confirmatory when adduction with inspiration is visualized, with or without provocation maneuvers [9]. Unfortunately, this is not always present and is not considered necessary for making the diagnosis [6].

Treatment options for PVFM include management of comorbid conditions, education, counselling, reassurance, and respiratory retraining. This includes introducing breathing techniques such asa jaw thrust, pursed lip breathing, nose to mouth breathing, panting and sniffing [10–14]. It is hypothesized that these specific techniques interrupt irregular respiratory spasms or patterns [7]. This allows familiar neurologic signals to reengage and relax the vocal folds [7]. Visual biofeedback involves allowing patients to visualize their own breathing patterns during routine flexible nasolaryngoscopy so that patients can modify their own breathing. This form of behavioural therapy gives patients techniques and strategies that can be practiced at home and used during acute attacks to improve their breathing [7, 10].

While multidisciplinary assessment and treatment of PVFM is ideal, this is not always possible. Access to timely specialized speech language pathology (SLP) services can be difficult in many places in Canada, resulting in long wait-times and delays in management. At our institution, wait-times for respiratory retraining therapy with speech language pathology is typically 6 to 9 months. This provided an opportunity to assess the effectiveness of surgeon performed visual biofeedback in the initial management of PVFM. While visual biofeedback has anecdotal effectiveness as an adjunct treatment of PVFM in the otolaryngology and speech language pathology community [15], few prospective studies exist.

## Methods

Institutional review board approval was obtained from the Human Research Ethics Board at the University of Alberta (Pro00076731).

### Patient population

All new patients referred for possible PVFM to a tertiary care laryngology practice between January 1st, 2018 to February 1st, 2020 were approached for participation in the study. Routine history, physical examination, and flexible in-office laryngoscopy was performed in all patients. Patients were excluded from the study if they had known refractory PVFM or were undergoing active SLP therapy. Additional exclusion criteria included patients in acute airway distress or those with untreated laryngeal, airway or lung pathologies. Patients with otherwise normal laryngoscopy underwent provocation maneuvers to elicit inspiratory adduction, but the absence of positive findings did not exclude the diagnosis of PVFM, which is in keeping with the current literature [16].

### Intervention

After topicalization of the nasal cavity with 1% lidocaine and 0.05% otrivin 50:50 mixture, surgeon-performed visual biofeedback was administered with continuous laryngoscopy performed using a distal-chip flexible rhinolaryngoscope. The following intervention was provided: explanation of normal laryngeal anatomy and breathing physiology, performance of laryngeal release exercises (e.g. rapid nasal breathing, pursed-lip breathing, etc.) [10, 13, 14]. Patients visualized their larynx throughout and were encouraged to palpate their anterior neck to recognize laryngeal tension, posture, and breathe control during laryngeal release exercises. Follow-up at 3 months and one-year was offered to all patients. Telephone follow-up was performed with patients who could not attend in-clinic follow-up due to geographic distance or patient-specific factors.

### Data collection

Baseline basic demographic information was collected including age, sex, and comorbidities. Specific information such as concurrent diagnosis of asthma, psychiatric illnesses, and smoking status were collected. Specific triggers for PVFM or laryngospasm episodes were also recorded, including exercise, acid reflux, upper respiratory tract infections, and environmental exposures.

### Primary outcome measure

The primary outcome measure was Dyspnea Index score pre- and 3 months post-intervention. The Dyspnea Index Score is a validated tool to measure subjective dyspnea symptom burden and has been previously validated by PVFM patients [17]. It is simple to administer, provides a measure of overall symptom severity, and can be used to monitor treatment progress [17]. The questionnaire was administered to patients in person at each the initial visit and at each follow-up.

### Secondary outcome measures

Our secondary outcome was change in patient reported use of inhaled bronchodilators and corticosteroids pre- and 3 months post-intervention. At baseline and follow-up, we recorded the patients’ reported weekly use of these medications in the preceding one-month time period.

### Sample size calculation

A previous study determined that a change in Dyspnea Index of 8 is considered the minimally clinically important difference (MCID) [17]. Using a power of 0.8 and alpha-error of 0.05, at least 16 patients with pre- and post-intervention data would be needed.

### Data analysis

Statistical analysis was carried out using SPSS (Statistical Package for the Social Sciences, Version 25, IBM Corp, Chicago, USA). Basic descriptive and frequency statistics were performed for patient demographics, comorbidities, and presenting symptoms. Paired sample t-test was used to compare pre- and post-intervention Dyspnea Index scores.

Wilcoxon signed-rank test to compare the differences in bronchodilator and corticosteroid use pre- and post-intervention at both are considered non-parametric, discrete data.

## Results

**Fig. 1:**
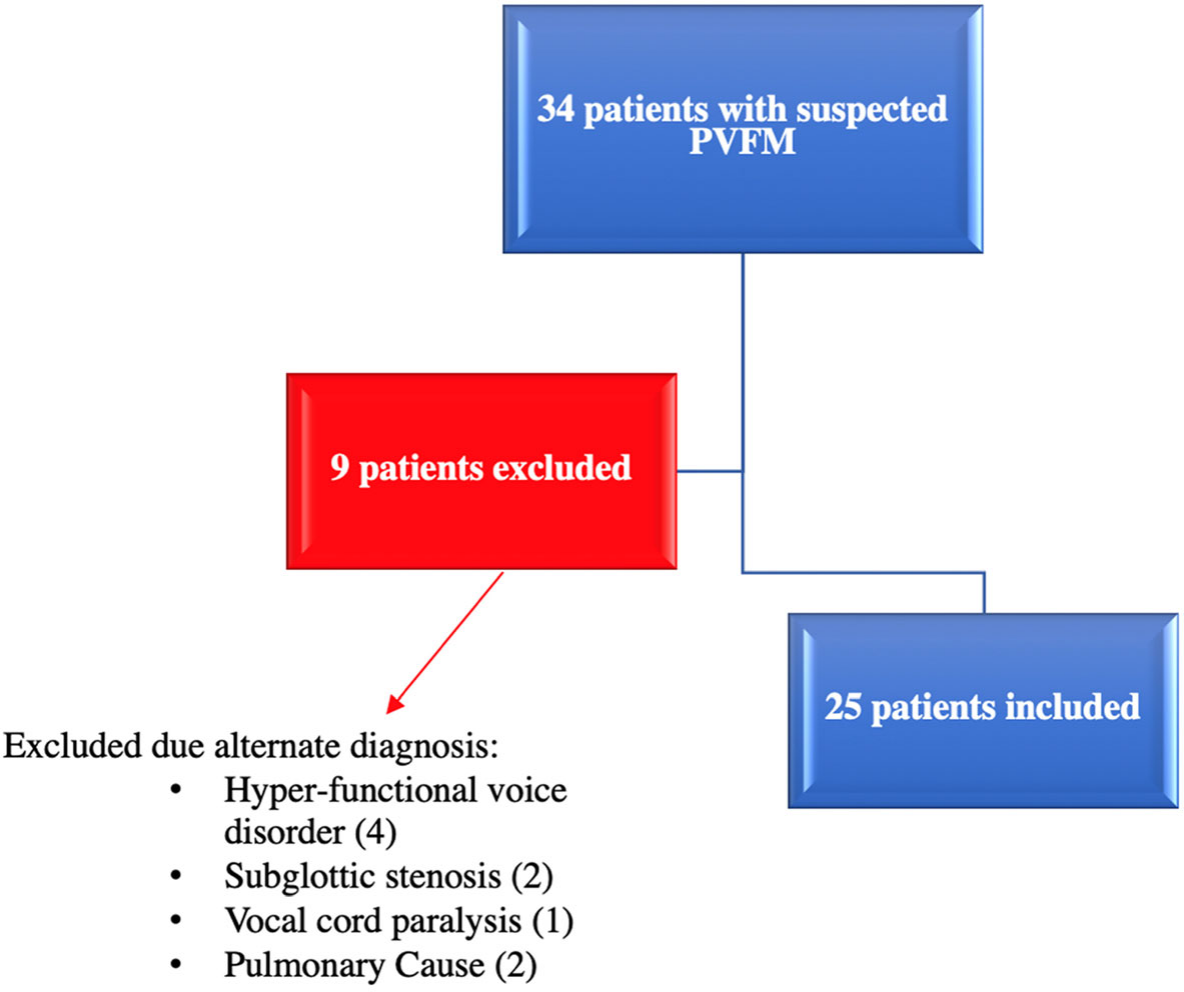
Flow Chart of patient recruitment

Thirty-four patients were referred for possible PVFM during the study period. Due to local referral patterns, nearly all the patients were referred by Pulmonology due to suggestive symptoms and/or the presence of inspiratory and expiratory flow restriction on pulmonary function tests (PFTs). Nine of these patients were excluded from our study due to presence of alternate diagnosis to explain their symptoms after clinical assessment. This included laryngeal pathologies such as functional voice disorders, subglottic stenosis, unilateral vocal cord paralysis and untreated lung pathology (Fig. 1).

Demographic information and relevant clinical information of the remaining 25 patients are summarized in Table 1. Twelve patients (48%) had a diagnosis of well-controlled asthma at the time of assessment based on Pulmonology assessment. Thirteen patients had a comorbid psychiatric diagnosis (e.G. *major* depression, anxiety disorder, or bulimia nervosa). Typical triggers of PVFM reported by patients included gastroesophageal reflux disease (GERD), exercise, upper respiratory tract infections (URTIs), speaking and cold air (Table 1).

**Table 1:** Summary of Patient Demographics

	N	Percentage
**Sex**		
Males	7	28%
Females	18	72%
**Mean Age ± SD (Range) years**	36.9 ± 14.4 (17.0 to 67.0)	
**Respiratory Diagnosis**		
Asthma	12	48%
**Psychiatric Diagnosis**		
Depression	7	28%
Anxiety	6	24%
Other	2	8%
**Triggers**		
Environmental	13	52%
Smoke	6	24%
Exercise	3	12%
Speaking	4	16%
URTI	2	8%
Cold Air	1	4%
Emotional stress	1	4%
GERD	1	4%

**Fig. 2:**
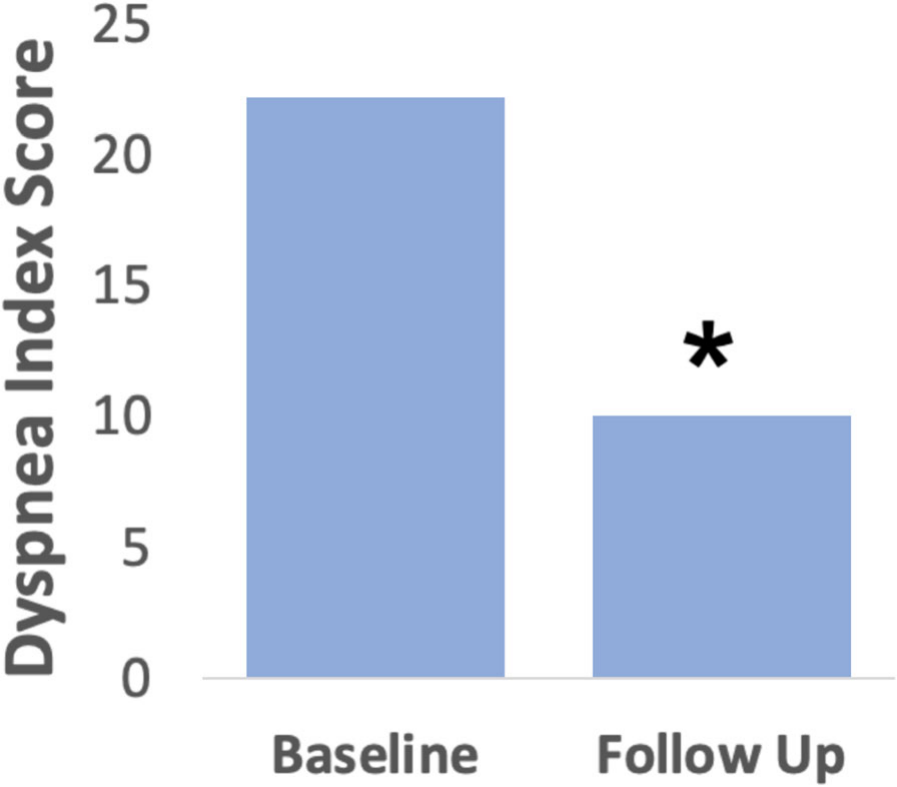
Dyspnea index paired sample t-test (*n* = 17) (t = − 6.02, *p* < 0.001)

**Fig. 3:**
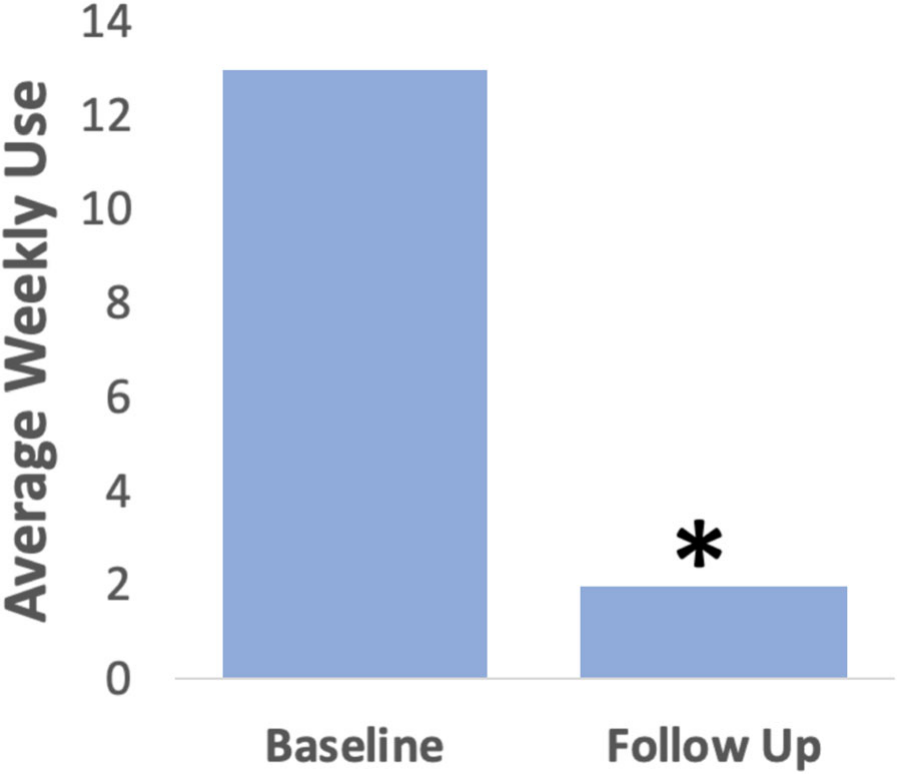
Bronchodilator use from baseline to follow-up (*n* = 13) (Z = − 2.934, *p* = 0.003)

Of the 25 patients, 17 completed the Dyspnea Index questionnaire pre- and 3 months post-intervention, with statistically significant reduction in scores (*p* < 0.001) and a mean difference of 12.1 (Fig. 2). Wilcoxon signed rank test was performed on thirteen patients who reported using bronchodilators pre-intervention. Our results showed a significant decrease in average bronchodilator use at 3 months post-intervention (Z = − 2.934, *p* = 0.003) (Fig. 3). Only two patients reported use of inhaled corticosteroids pre-intervention and both reported no use post-intervention. One patient was referred for further assessment by speech language pathology for ongoing refractory symptoms. Eight patients did not attend an in-person follow-up at 3 months, and of these, six patients reported no residual symptoms by telephone follow-up. While all patients were contacted for a one-year follow-up, twenty-three declined due to lack of symptoms and two patients could not be reached.

## Discussion

We conducted a non-randomized, prospective study examining the use of surgeon-performed visual biofeedback in the management of PVFM patients. Our study provides support for its use in the initial management of patients presenting non-acutely, particularly in settings where specialized speech language pathology services are not readily available or delayed.

A variety of treatment options currently exist for the treatment of PVFM. This includes pharmacotherapy, counselling, laryngeal Botulinum toxin injection, education and behavioural therapy with respiratory retraining [13, 18]. Treatment of concurrent medical conditions is crucial as it may exacerbate PVFM symptoms [19]. Visual biofeedback is easy to administer at the time of patient assessment by the Otolaryngologist. In addition to providing immediate reassurance, basic teaching regarding laryngeal anatomy and breathing techniques are provided immediately to the patient. It builds on existing studies demonstrating the efficacy of respiratory retraining and laryngeal control therapies to reduce PVFM-related symptoms [13, 18–20]. Dr. Konstantin Buteyko first described these techniques for asthma patients, including a focus on nasal-breathing, breath-holding, and relaxation in the 1950’s and multiple subsequent studies have demonstrated reductions in dyspnea symptoms, inhaler use and improved quality of life in asthmatic patients [21–26]. Later on, Blager, Gay & Wood in 1988 described the first behavioural interventions for patients with habitual cough and vocal cord dysfunction, which is now largely used across the world [13].

There is a complex relationship between asthma and PVFM, as the two diagnosis are often confused and co-exist. It has been reported that as many as 30 to 50% of patients with a diagnosis of asthma have a coexisting diagnosis of PVFM [27]. Almost half of our patients were diagnosed with asthma by a pulmonologist or primary care physician. It is unclear if the reduction in bronchodilator and inhaled corticosteroid use in our study is due to improved patient self-control of acute PVFM episodes versus improved self-management of asthma. However, our results are in line with other studies showing that improved PVFM control can reduce asthma medication use [18, 19]. Kramer et al. demonstrated that patients with PVFM who received education and a course of laryngeal control therapy by a licensed speech language pathologist had reductions in the total use of asthma pharmacotherapy, including short and long acting beta-agonists, inhaled and oral corticosteroids, leukotriene inhibitors and inhaled anticholinergic [18].

### Study limitations

There are a few limitations to our study. The Dyspnea Index score is a global subjective dyspnea scale and was used as a surrogate for subjective PVFM control. Information regarding the actual change in number of episodes was not recorded. Our secondary outcomes of change in bronchodilator and inhaled corticosteroid use relies on patient memory and is subject to recall bias. In addition, this study focused on short-term follow-up of 3 months. Since most patients declined one-year follow-up, long-term outcomes data and recidivism over time could not be assessed. Finally, our study focuses on new PVFM patients rather than difficult-to treat or refractory patients. Specific populations, including elite athletes often have persistent symptoms despite routine therapeutic trials [28]. A recent study reported the use of the Olin EILOBI (Exercise Induced Laryngeal Obstruction Biphasic Inspiration) breathing techniques to reduce dyspneic symptoms in this specific patient population [29].

While our study did not record measures of healthcare utilization, such as visits to the emergency room, or previous intubations, this is an avenue of further research. Future studies could examine the potential reductions in healthcare costs due to avoidance of emergency room access, intubations and potentially intensive care unit stay.

## Conclusions

Visual biofeedback shows a statistically and clinically significant reduction in symptoms and the use of asthma medication. Our study demonstrates an efficient and effective way of diagnosing and managing new patients with PVFM on the same day. We recommend visual biofeedback as a primary treatment for newly diagnosed PVFM patients.

## Abbreviations

DI: Dyspnea Index
EILOBI: Exercise Induced Laryngeal Obstruction Biphasic Inspiration
MCID: Minimally clinically important difference
PVFM: Paradoxical vocal fold motion
PFT: Pulmonary function test
SLP: Speech Language Pathology
